# Esculetin Attenuates Inflammation and Fibrosis to Prevent AKI-to-CKD Transition in Adenine-Induced Renal Injury by Inhibiting the EGFR/SRC/PI3K/AKT/NF-κB Signaling Axis

**DOI:** 10.3390/ph19040578

**Published:** 2026-04-03

**Authors:** Jianglong Chen, Bin Xia, Rujie Zhou, Yunfei Cui, Yu Zhu, Meijia Chen, Jinhua Su, Jinhui Wang, Guang Li

**Affiliations:** 1 College of Pharmacy, Heilongjiang University of Chinese Medicine, Harbin 150006, China; cjl2913183067@163.com (J.C.); 15599331865@163.com (R.Z.); 2 Yunnan Key Laboratory of Southern Medicine Utilization, Institute of Medicinal Plant Development, Chinese Academy of Medical Sciences & Peking Union Medical College, Jinghong 666100, China; 2913183067@qq.com (B.X.); 719318oyf@163.com (Y.C.); 2848342113@qq.com (Y.Z.); 1851297024@qq.com (M.C.); 3 Xishuangbanna Dai Autonomous Prefecture Institute of Drug Supervision and Inspection, Jinghong 666100, China; 17683952849@163.com; 4 Xishuangbanna Dai Medicine Research Institute Co., Ltd., Jinghong 666100, China; penaltyow@outlook.com

**Keywords:** chronic kidney disease, esculetin, network pharmacology, transcriptomics, NF-κB, AKI-to-CKD transition, prophylactic intervention

## Abstract

**Background:** Chronic kidney disease (CKD) is characterized by irreversible structural damage and functional deterioration of the kidneys. Esculetin (ES), with its anti-inflammatory, antioxidant, and immunomodulatory activities, shows potential in delaying renal function decline. This study aimed to investigate the protective effect of ES on adenine-induced CKD in mice and its underlying molecular mechanism, with a focus on its role in preventing the transition from acute kidney injury (AKI) to CKD. **Methods:** A AKI-to-CKD transition mice model was established by feeding mice a 0.2% adenine diet, and ES (30, 60 mg/kg) was co-administered for 4 weeks as a prophylactic intervention. Serum creatinine (SCr), blood urea nitrogen (BUN), and renal histopathology (HE, Masson, IHC) were evaluated to assess renal injury. Network pharmacology and transcriptomics were combined to screen the targets, and Western blot was used to verify the signaling pathways. **Results:** ES significantly reduced SCr and BUN levels in CKD mice and alleviated renal tubular dilation and inflammatory infiltration. ES decreased pro-inflammatory factors (IL-1β, IL-6, TNF-α) and MDA levels and enhanced SOD activity. Additionally, ES inhibited renal interstitial collagen deposition and reversed epithelial–mesenchymal transition (EMT) by upregulating E-cadherin and downregulating α-SMA levels. Mechanism studies confirmed that ES significantly inhibited the phosphorylation levels of p-EGFR, p-SRC, p-PI3K, p-AKT, and p-p65 in renal tissues. **Conclusions:** ES effectively inhibits inflammation, oxidative stress, and fibrosis by modulating the EGFR/SRC/PI3K/AKT/NF-κB signaling axis, thereby preventing the AKI-to-CKD transition in the adenine-induced renal injury model and alleviating the progression of chronic renal damage.

## 1. Introduction

Chronic Kidney Disease (CKD) is a comprehensive disease characterized by progressive damage to the structure of the kidneys and irreversible deterioration of renal function [1]. The incidence of CKD among the global adult population has exceeded 10%, and it is on the rise each year due to the aging population and the prevalence of diseases such as diabetes and hypertension [2]. CKD has insidious onset, and early symptoms lack specificity. Once it progresses to the middle and late stages, it is often accompanied by irreversible renal fibrosis and may eventually develop into end-stage renal disease (ESKD), at which point patients need to rely on dialysis or kidney transplantation to maintain life, imposing a heavy economic and medical burden on families and the social medical system [3]. Currently, the intervention of CKD mainly focuses on controlling underlying diseases such as blood pressure, blood sugar, and blood lipids to slow down the progression of the disease, but the efficacy effect is often unsatisfactory [4]. Therefore, the intervention and prevention of CKD has become a global clinical challenge, and finding safe, effective, and low-cost preventive and interventional strategies has become an urgent need for current research.

Esculetin (ES) is a natural coumarin compound extracted from the traditional Chinese medicine Cortex Fraxini. Previous studies have confirmed that it possesses pharmacological activities such as anti-inflammation, antioxidation, and immunomodulation [5]. In the field of renal injury protection, esculetin has shown significant effects [6,7,8,9,10]. For lupus nephritis (an autoimmune kidney disease that can progress to CKD), it alleviates chronic kidney tissue damage by inhibiting abnormal complement activation and enhancing the Nrf2 signaling pathway, thereby delaying progression to the terminal stage of CKD [6]. For diabetic nephropathy (the leading cause of CKD), it can suppress long-term oxidative stress in kidney tissues, regulate fibrotic pathways (reducing collagen deposition and fibronectin expression), and slow down the progression of streptozotocin-induced diabetic kidney injury in mice, protecting glomerular filtration function [7]. For acute kidney injury and diabetes comorbidity, its combination with fraxetin can promote mitochondrial autophagy and inhibit the release of chronic pro-inflammatory factors such as TNF-α, reducing the risk of developing CKD [8,9,10]. In summary, esculetin can act on the main causes of CKD (diabetes, autoimmunity) and key pathological processes (chronic inflammation, fibrosis, oxidative stress), with low toxicity and effective protection of long-term renal function in animal experiments. It has significant research value and application potential in the field of delaying the course of chronic kidney disease and preventing renal injury progression, and it is expected to become a candidate or adjuvant drug for the prevention and intervention of CKD in the future.

Although studies have shown that esculetin is a renal protective agent for CKD, most of the related research has focused on acute kidney injury and diabetic kidney injury, with the majority consisting of phenotype correlation studies without involving the verification of specific pathways and chronic CKD models [5,6,7,8,9,10]. However, this study used a adenine-induced renal injury model (a classic AKI-to-CKD transition model) different from previous studies, and ES was co-administered with adenine as a prophylactic intervention. The study focused on specific pathways through network pharmacology and transcriptome for verification, aiming to clarify the effect of ES on preventing the AKI-to-CKD transition and its underlying mechanism.

## 2. Results

### 2.1. Results of Network Pharmacology Analysis

**Figure 1:**
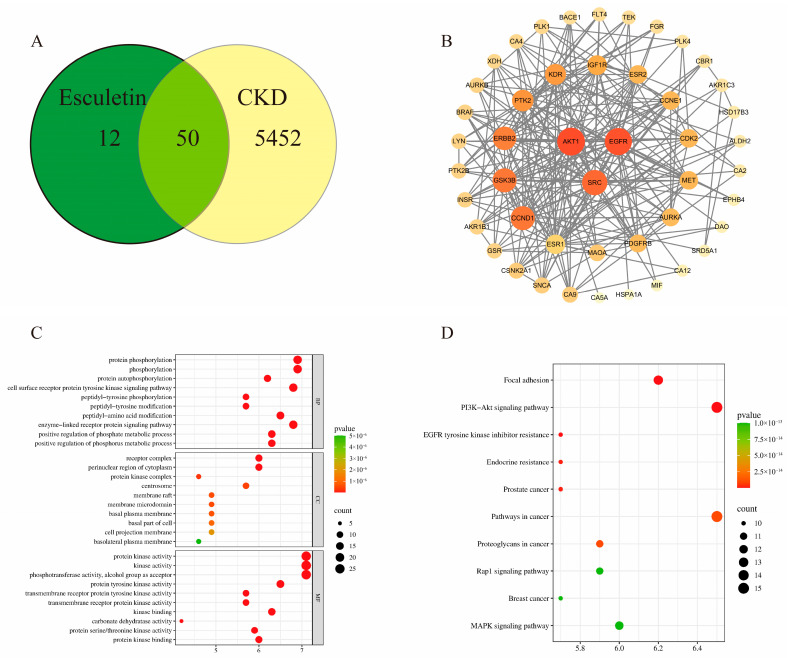
Results of network pharmacology analysis: (**A**) Venn diagram of intersection targets; (**B**) PPI network diagram of intersection targets; (**C**) GO enrichment analysis; (**D**) KEGG enrichment analysis.

From the Swiss Target Prediction database, 62 non-repetitive target components of esculetin were predicted. From the Gene Cards database, 5501 non-repetitive CKD-related target points were retrieved. The intersection analysis of these two sets yielded 50 drug–disease intersection targets (Figure 1A). The PPI network was constructed through the STRING database and visualized by Cytoscape. Based on the combined scores of connectivity, betweenness centrality, and closeness centrality, the top three core targets were selected: AKT1, SRC, and EGFR (Figure 1B). GO functional enrichment analysis indicated that the intersection targets were mainly involved in biological processes such as protein phosphorylation and cell surface receptor protein tyrosine kinase signaling pathways (Figure 1C). KEGG pathway enrichment analysis identified core pathways, including PI3K-Akt, EGFR, MAPK, and Rap1 (Figure 1D).

### 2.2. Results of Transcriptome Analysis

**Figure 2:**
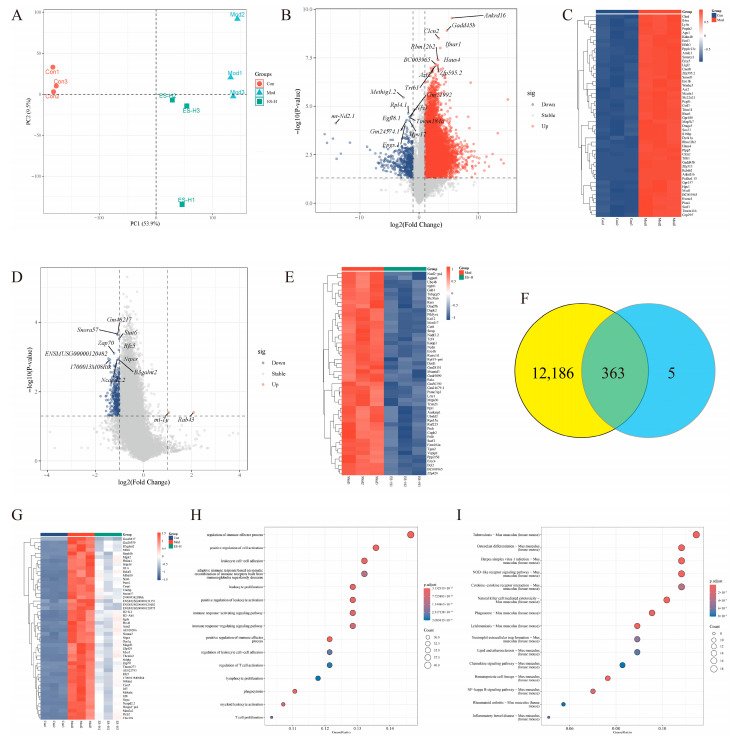
Results of transcriptome analysis: (**A**) PCA analysis; (**B**) volcano plot of Con vs. Mod; (**C**) heatmap of Con vs. Mod; (**D**) volcano plot of Mod vs. ES-H; (**E**) heatmap of Mod vs. ES-H; (**F**) Venn diagram of intersection analysis of differentially expressed genes, Yellow: Con vs. Mod, Blue: Mod vs. ES-H; (**G**) heatmap of common differentially expressed genes; (**H**) GO enrichment analysis of common differentially expressed genes; (**I**) KEGG enrichment analysis of common differentially expressed genes.

To further explore the mechanism of esculetin in protecting mice from adenine-induced renal injury and preventing AKI-to-CKD transition, transcriptome analysis was conducted. PCA analysis showed that the samples of the Con (control) group, Mod (model) group, and ES-H (Esculetin-High) group were clearly distributed, indicating significant differences at the transcriptional level (Figure 2A). With FDR < 0.05 and |log_2_FC| ≥ 1 as the screening threshold, differentially expressed genes (DEGs) were screened for each group of samples. The results are as follows: A total of 12,549 DEGs were screened out in the Con vs. Mod group, including 12,050 upregulated genes and 499 downregulated genes (Figure 2B,C). A total of 368 DEGs were screened out in the Mod vs. ES-H group, including 2 upregulated genes and 366 downregulated genes (Figure 2D,E). Visualization of differentially expressed genes: In the volcano plot, red dots represent upregulated DEGs, green dots represent downregulated DEGs, and gray dots represent non-differentially expressed genes. The significant differentially expressed genes in each group are mainly distributed at both ends of the volcano plot, indicating that the expression changes and statistical significance of the screened DEGs meet the requirements. The heatmap of the top 50 DEGs shows that the gene expression patterns of samples within the same group are highly consistent, while the gene expression patterns of samples between groups are significantly different. This can clearly distinguish different intervention groups, further verifying the reliability of the differential gene screening. Intersection analysis of differentially expressed genes: There were 363 common DEGs between the Con vs. Mod group and the Mod vs. ES-H group (Figure 2F), and the expression of these 363 genes was significantly upregulated in the Mod group and significantly downregulated in the ES-H group (Figure 2G). GO and KEGG enrichment analysis were performed on the 363 common DEGs. GO functional enrichment analysis showed that the common DEGs were mainly involved in the regulation of immune effector processes, activation of immune response signaling pathways, and regulation of T cell activation (Figure 2H). KEGG pathway enrichment analysis identified core pathways, such as NOD-like receptor, Chemokine, and NF-κB (Figure 2I).

### 2.3. Combined Analysis of Transcriptome and Network Pharmacology

The network pharmacology analysis revealed that the core targets of esculetin mainly concentrated on kinases such as EGFR and SRC, and the results of GO and KEGG enrichment were highly concentrated on protein phosphorylation, cell surface receptor protein tyrosine kinase signaling pathways, and upstream signal transduction pathways such as PI3K-Akt. This suggests that esculetin may mainly exert its prophylactic intervention effect by targeting and regulating the activity of upstream kinases. Transcriptome analysis showed that 363 common differentially expressed genes (upregulated in the Mod group and downregulated in the ES-H group) were mainly enriched in biological processes, such as immune effect regulation and T cell activation, as well as typical inflammation-related KEGG pathways, such as NF-κB, Chemokine, and NOD-like receptor. The transcriptome results visually depict the excessive activation of the local immune-inflammatory microenvironment in the kidney under the pathological state of adenine-induced early renal injury and the significant reversal effect of esculetin on this process, which is the key to preventing the subsequent AKI-to-CKD transition.

**Figure 3:**
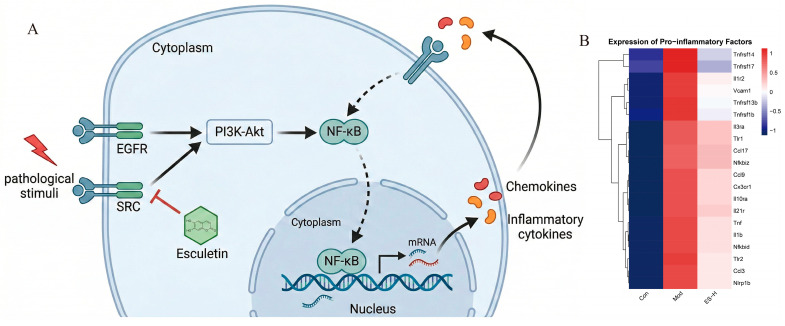
Combined analysis of transcriptome and network pharmacology: (**A**) “Target-pathway-phenotype” cascade regulation; (**B**) mRNA expression of inflammatory factors.

Combining the results of the two omics, this study constructed a “target-pathway-phenotype” cascade regulatory network of esculetin in the prevention of AKI-to-CKD transition in adenine-induced renal injury (Figure 3A). During the onset of adenine-induced renal injury (AKI phase), the kidney tissue may be pathologically stimulated, which may lead to the abnormal activation of upstream receptors and kinases such as EGFR and SRC, and then transmit signals downstream to activate the PI3K-Akt pathway [11]. The excessive activation of these upstream signals ultimately converges on the downstream core transcription factor NF-κB. The activation of NF-κB and its nuclear translocation directly drive the large-scale transcriptional expression of a large number of chemokines and immune-inflammatory related genes [12]. In this microenvironment, key pro-inflammatory factors may also be activated, forming a positive feedback loop with NF-κB, exacerbating kidney damage and promoting the AKI-to-CKD transition [13]. The combined analysis indicated that esculetin could intervene in the upstream kinase targets, such as EGFR and SRC, block the downward cascade transmission of pathological signals, and effectively inhibit the activity of core transcription pathways such as NF-κB. This ultimately widely downregulates the expression of downstream inflammatory factors at the transcriptional level (Figure 3B), exerting a protective effect in preventing the AKI-to-CKD transition and alleviating chronic renal damage.

### 2.4. Protective Efficacy of ES on Mice with Adenine-Induced Renal Injury

#### 2.4.1. ES Can Effectively Alleviate the Progression of Renal Injury and Prevent AKI-to-CKD Transition in Mice

**Figure 4:**
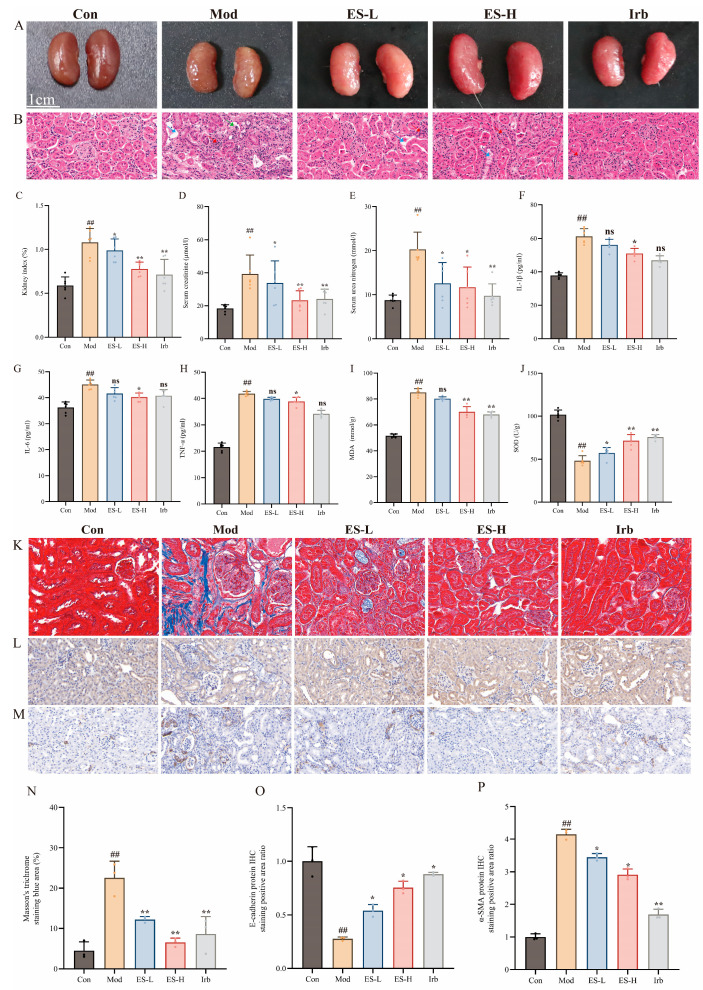
Evaluation of the protective effect of ES on adenine-induced renal injury mice: (**A**) gross observation of the kidney; (**B**) HE staining (×200; red arrows denote inflammatory infiltration, green arrows denote glomerular injury, and blue arrows denote renal tubular injury); (**C**) kidney index (*n* = 6); (**D**) serum creatinine (*n* = 6); (**E**) serum urea nitrogen (*n* = 6); (**F**) IL-1β (*n* = 6); (**G**) IL-6 (*n* = 6); (**H**) TNF-α (*n* = 6); (**I**) MDA (*n* = 6); (**J**) SOD (*n* = 6); (**K**) Masson staining (×200); (**L**) E-cadherin immunohistochemical staining (×200); (**M**) α-SMA immunohistochemical staining (×200); (**N**) statistical analysis of positive area in Masson staining (*n* = 3); (**O**) statistical analysis of positive area in E-cadherin immunohistochemical staining (*n* = 3); (**P**) statistical analysis of positive area in α-SMA immunohistochemical staining (*n* = 3). Compared with the Con group, ## indicates *p* < 0.01; compared with the Mod group, * indicates *p* < 0.05, ** indicates *p* < 0.01, and ns indicates *p* > 0.05.

The adenine-induced renal injury mice model exhibited obvious renal damage. Macroscopic observation revealed that the kidneys of the Mod group were pale, firm in texture, and covered with dense white granules. ES prophylactic intervention significantly improved these morphological pathological changes (Figure 4A). Histopathological HE staining (Figure 4B) further confirmed that the Mod group had severe glomerular atrophy, tubular dilation, and inflammatory cell infiltration, all of which were effectively alleviated in the ES intervention groups. To evaluate the renal protective effect of ES on renal function in renal injury mice, the kidney index and SCr and BUN were measured. The results show (Figure 4C–E) that compared with the Con group, the kidney index of the Mod group significantly increased from 5.88 ± 0.98% to 10.80 ± 1.59% (*p* < 0.01), indicating severe compensatory hypertrophy of the kidneys. Meanwhile, the serum SCr and BUN levels in the model group were 39.95 ± 6.87 umol/L and 17.62 ± 1.09 mmol/L, respectively, which were significantly higher than those in the normal group (SCr: 18.33 ± 2.32 µmol/L; BUN: 8.80 ± 1.13 mmol/L) (*p* < 0.01), suggesting successful modeling and severe renal function impairment in the mice. After ES intervention, the renal function indicators in all intervention groups were improved to varying degrees. Specifically, the kidney index in the high-dose group (ES-H) was significantly reduced to 7.77 ± 0.79% (*p* < 0.01). In terms of biochemical indicators, ES at all doses could effectively reduce SCr and BUN levels. Specifically, the high-dose group significantly reduced SCr to 26.17 ± 3.07 µmol/L (*p* < 0.01) and BUN to 13.79 ± 1.25 mmol/L (*p* < 0.01), with efficacy comparable to the positive control (irbesartan, Irb) group (BUN: 11.62 ± 1.10 mmol/L, *p* < 0.01). These results indicate that ES can effectively alleviate the progression of adenine-induced AKI and prevent the subsequent AKI-to-CKD transition in mice.

#### 2.4.2. ES Can Improve the Inflammatory Response and Oxidative Stress in Mice with Adenine-Induced Renal Injury

To evaluate ES’s regulatory effect on inflammatory responses and oxidative stress in adenine-induced renal injury, we detected the relevant biochemical indicators in the serum and renal tissues of mice. The results show that compared with Con, the levels of pro-inflammatory factors in Mod mice were significantly increased (Figure 4F–H), among which IL-1β rose to 61.18 ± 4.502 pg/mL (*p* < 0.01), IL-6 rose to 45.08 ± 1.689 pg/mL (*p* < 0.01), and TNF-α rose to 41.85 ± 0.8456 pg/mL (*p* < 0.01), indicating that adenine induction led to a high inflammatory state in mice. In terms of oxidative stress (Figure 4I,J), the lipid peroxidation product MDA in the renal tissues of the model group significantly accumulated to 85.12 ± 2.88 mmol/g (*p* < 0.01), while the key indicator of the antioxidant defense system, SOD (48.23 ± 5.822 U/g), was significantly downregulated compared with the control group (*p* < 0.01), suggesting that the ability of the kidneys to clear oxygen free radicals was severely impaired. After intervention with high-dose ES (ES-H), the above pathological indicators were significantly improved: IL-1β, IL-6, and TNF-α levels were reduced to 47.25 ± 6.254 pg/mL, 39.63 ± 3.884 pg/mL, and 32.15 ± 3.614 pg/mL, respectively (all *p* < 0.05). At the same time, oxidative stress damage was significantly alleviated, as indicated by a significant decrease in MDA content (74.02 ± 4.797 mmol/g, *p* < 0.01), and the activity of endogenous antioxidant enzymes was effectively restored, with SOD rising to 65.6 ± 9.846 U/g (*p* < 0.01). The above results suggest that ES can effectively improve the inflammatory response and oxidative stress damage in adenine-induced renal injury mice, thereby inhibiting the AKI-to-CKD transition by blocking the key pathological drivers of chronic renal damage.

#### 2.4.3. ES Can Significantly Alleviate Renal Fibrosis in Mice with Adenine-Induced Renal Injury

To evaluate the effect of ES on renal fibrosis in CKD, this study first observed the pathological changes in the renal tissues of mice by Masson staining. The results show (Figure 4K,N) that compared with Con, obvious collagen fiber deposition was observed in the renal interstitium of Mod mice, and the proportion of blue-stained area was significantly increased (*p* < 0.01). After intervention with ES (low and high doses) and Irb, the collagen deposition in the renal tissues was significantly alleviated, and the blue-stained area was significantly reduced compared with the model group (*p* < 0.01). Subsequently, the expression levels of fibrosis-related marker proteins α-SMA and E-cadherin were detected by immunohistochemical staining (IHC). In the in vivo experiments (Figure 4L,M,O,P), the protein level of the epithelial cell marker E-cadherin in the renal tissues of the model group was significantly decreased compared with the control group (*p* < 0.01), while the level of the mesenchymal marker α-SMA was abnormally increased (*p* < 0.01), suggesting a significant EMT process. ES intervention significantly reversed this trend, upregulated the expression of E-cadherin (*p* < 0.01), and inhibited the overexpression of α-SMA (*p* < 0.01), showing a certain dose dependence. In conclusion, in the adenine-induced CKD mice model, ES effectively inhibited renal collagen deposition and epithelial–mesenchymal transition by regulating the expression of fibrosis marker proteins, thereby alleviating the early fibrotic changes and preventing the AKI-to-CKD transition.

### 2.5. Verification of the Mechanism of ES in Preventing AKI-to-CKD Transition in Adenine-Induced Renal Injury

**Figure 5:**
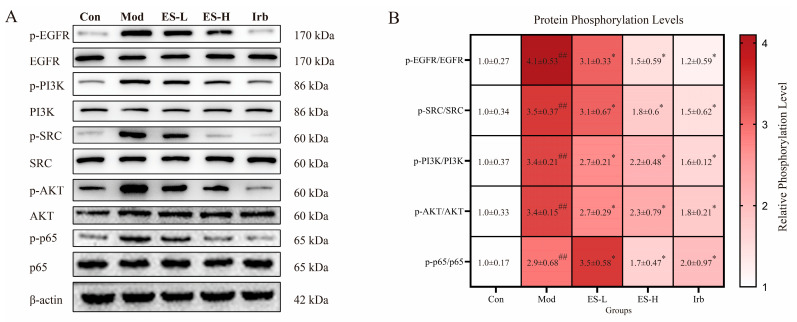
Verification of the mechanism of ES in improving adenine-induced renal injury mice. (**A**) Immunoblotting images of p-EGFR, p-SRC, p-PI3K, p-AKT, and p-p65. (**B**) Statistical graphs of p-EGFR, p-SRC, p-PI3K, p-AKT, and p-p65 (*n* = 3). Compared with the Con group, ## indicates *p* < 0.01; compared with the Mod group, * indicates *p* < 0.05.

Based on the results of the combined analysis, we detected the expression of p-EGFR, p-SRC, p-PI3K, p-AKT, and p-p65 in the renal tissues of CKD mice. The results show (Figure 5) that compared with the Con group, the expression of p-EGFR, p-SRC, p-PI3K, p-AKT, and p-p65 in the renal tissues of the Mod group was significantly increased (*p* < 0.01). After intervention with ES (low and high doses), the expression of p-EGFR, p-SRC, p-PI3K, p-AKT, and p-p65 was significantly decreased (*p* < 0.05).

## 3. Discussion

CKD has become a global public health challenge, characterized by progressive loss of nephrons and irreversible glomerular sclerosis and interstitial fibrosis [1]. Despite the availability of multiple clinical intervention methods, there is still a lack of effective drugs targeting the core pathological process of AKI-to-CKD transition—the inflammatory and fibrotic cascade reaction [4]. Esculetin (ES), a natural coumarin compound derived from Fraxinus, has attracted much attention due to its excellent anti-inflammatory and antioxidant biological activities [5]. In this study, by establishing an adenine-induced renal injury model and performing prophylactic co-administration of ES with adenine, we systematically confirmed that ES could significantly reduce SCr and BUN levels and alleviate renal tissue pathological damage. More importantly, through the integration of network pharmacology, transcriptome sequencing, and molecular biology verification, this study, for the first time, revealed the deep mechanism by which ES exerts renal protective effects by inhibiting the EGFR/SRC/PI3K/AKT/NF-κB signaling axis, providing a solid theoretical basis for the application of ES in the prevention of AKI-to-CKD transition from basic research to clinical practice.

As a potential clinical drug, safety is the primary consideration. Acute toxicity experiments in mice have shown that the oral LD50 (median lethal dose) of esculetin is greater than 2000 mg/kg, while its effective pharmacological dose is usually between 10 and 50 mg/kg [14,15]. This indicates that esculetin has a relatively wide therapeutic window and is relatively safe for oral administration.

One of the significant innovations of this study lies in using the adenine-induced AKI-to-CKD transition model to explore the mechanism of ES in preventing the progression of renal injury in mice. Unlike previous studies that have mostly focused on acute kidney injury (AKI) or streptozotocin-induced diabetic nephropathy (DN) models, the adenine model can more accurately simulate the typical pathological process of clinical AKI-to-CKD transition, including metabolic disorders, significant interstitial inflammatory infiltration, and extensive renal fibrosis [16]. The previous literature has reported the protective effect of ES in lupus nephritis and DN, but it has mostly been limited to phenotype observations under specific etiologies [5,6,7,8,9,10]. In this study, ES was observed to systematically improve renal function in the adenine-induced model, demonstrating its universal protective value in non-metabolic, AKI-induced chronic progressive renal injury. Additionally, the significant performance of ES in reducing the proportion of blue-stained area in Masson staining of renal tissue indicates its role in reversing early renal interstitial fibrosis, a key stage in the AKI-to-CKD transition.

At the level of mechanism exploration, this study did not confine itself to a single renal protective efficacy evaluation but adopted a research approach of “computational prediction + high-throughput screening + experimental verification” [17]. By using network pharmacology to screen out the potential targets of ES and combining transcriptome sequencing to analyze the differentially expressed genes in the kidney tissues of mice with adenine-induced renal injury, this dual verification strategy significantly enhanced the accuracy of target identification. The study found that signal molecules such as EGFR, SRC, PI3K, and AKT showed high correlation and significant expression differences in the renal injury model group. This networked research approach that bridges macroscopic renal protective efficacy to microscopic molecular interactions through systems biology methods overcomes the drawback of “vague mechanisms” in traditional Chinese medicine research and represents a significant methodological breakthrough in this study.

The core finding of this study is the significant inhibitory effect of ES on EGFR and its downstream signaling axis. EGFR, as a receptor tyrosine kinase, has been recognized as a key switch for triggering inflammation and fibrosis after kidney injury [18]. Inflammatory inhibition mechanism: Experimental data show that ES significantly reduces the expression of pro-inflammatory factors IL-1β, IL-6, and TNF-α. This is because ES inhibits the phosphorylation of EGFR/SRC, blocking the activation of the downstream PI3K/AKT pathway, and thereby restricting the nuclear translocation of the transcription factor NF-κB p65. As a core regulator of the inflammatory response, the suppression of NF-κB directly leads to the termination of the inflammatory cascade, thereby alleviating the continuous damage to the renal microenvironment [19]. Anti-fibrotic and antioxidant effects: This study observed that after ES treatment, the expression of the fibrosis marker α-SMA was downregulated, while the expression of E-cadherin was restored, suggesting that ES can inhibit the EMT of renal tubular epithelial cells. At the same time, ES effectively restored the activity of the endogenous antioxidant enzyme SOD in the body and reduced the content of lipid peroxidation product MDA. This indicates that the protective effect of ES is multi-dimensional, not only regulating protein expression through signaling pathways but also improving the oxidative stress state by eliminating oxygen free radicals.

Notably, esculetin has been reported to exhibit seemingly unfavorable ADMET properties for long-term pharmacological intervention, including a short half-life (~2 h) and low oral bioavailability (19%) in rats due to rapid glucuronidation [5,20], as well as rapid systemic elimination from the body [21]. These characteristics are generally considered adverse for the treatment of established chronic diseases that require sustained systemic drug exposure. However, these properties can be well reconciled with the positive protective effects observed in our study, which is closely related to the prophylactic intervention mode and AKI-phase intervention stage of this research. First, our study adopted a daily oral gavage administration mode for 4 consecutive weeks; although the systemic half-life of ES is short, repeated daily administration can maintain a stable local effective concentration of ES in renal tissues, which compensates for the deficiency of low systemic bioavailability and rapid elimination. Second, the glucuronidation of ES (the main metabolic pathway leading to low bioavailability [5]) does not completely inactivate its biological activity, and its glucuronide metabolites can still accumulate in renal tissues and exert anti-inflammatory and antioxidant effects, which is a common characteristic of natural coumarin compounds with renal targeting potential. Third, this study focused on the prophylactic intervention of the AKI phase in the AKI-to-CKD transition model rather than the treatment of established CKD with irreversible fibrosis. The AKI phase is an early pathological stage of renal injury, which only requires the inhibition of acute inflammatory and fibrotic initiation signals rather than sustained high-concentration systemic drug exposure; the local renal tissue effect of ES is sufficient to block the key pathological drivers of AKI-to-CKD transition. In addition, the pathological microenvironment of adenine-induced renal injury (e.g., inflammatory cell infiltration, increased vascular permeability) may promote the enrichment of ES in damaged renal tissues, further improving the local effective concentration and offsetting the influence of systemic ADMET properties.

In the experimental design, irbesartan was introduced as the positive control group. The results show that ES at high dose (ES-H) exhibited renal protective activity comparable to irbesartan in multiple indicators, especially in regulating inflammatory factors and the antioxidant defense system. Irbesartan mainly exerts its effect by antagonizing the angiotensin II receptor, while ES demonstrates a broader pharmacological activity spectrum [5,22]. Considering the complexity of the pathogenesis of AKI-to-CKD transition, the “multi-component, multi-target, multi-pathway” characteristics of ES give it a unique advantage in combination preventive intervention or as a second-line adjuvant preventive strategy.

## 4. Materials and Methods

### 4.1. Materials

Materials included esculetin (Adamas Reagent Co., Ltd., Shanghai, China), an HE staining kit, a Masson staining kit (Beijing Solarbio Science & Technology Co., Ltd., Beijing, China), serum creatinine, urea nitrogen (Wuhan Jilide Biotechnology Co., Ltd., Wuhan, China), SOD, MDA, IL-6, IL-1β, and TNF-α ELISA kits (Wuhan Sevewell Biotechnology Co., Ltd., Wuhan, China), and antibodies EGFR, p-EGFR, SRC, p-SRC, p-PI3K, PI3K, AKT, p-AKT, p-p65, p65.

### 4.2. Animal Experiments

#### 4.2.1. Animal Grouping and Administration

All animals were purchased from Beijing Sibeifu Biotechnology Co., Ltd. (Beijing, China) and were housed at the Yunnan Branch of the Institute of Medicinal Plants, Chinese Academy of Medical Sciences (12 h light, 12 h dark, 24 °C, 55–65% humidity). Thirty male C57BL/6J mice (6–8 weeks old, 20–25 g) were randomly divided into five groups (*n* = 6 per group). The sample size of 6 mice per group was a priori determined based on the sample size design of classic adenine-induced renal injury models [16,23] and pilot experiment results, which ensured over 80% statistical power for detecting significant differences in key outcome measures via one-way ANOVA. The sample size also complied with the 3R principles for animal experimentation. The randomization sequence was generated using the random number table function of GraphPad Prism 8.0.2, and the mice were assigned to five groups by ascending random numbers. Confounding factors were minimized by random arrangement of breeding cages, randomized operation order for administration and sample detection, and strict adherence to unified housing conditions, consistent reagents/equipment, and standardized experimental operations for all groups. There was a control group (standard diet + 10 mL/kg distilled water by gavage), an adenine model group (0.2% adenine diet + 10 mL/kg distilled water by gavage), an ES-L (Esculetin-Low) group (0.2% adenine diet + 30 mg/kg esculetin by gavage), an ES-H (Esculetin-High) group (0.2% adenine diet + 60 mg/kg esculetin by gavage), and an Irb (irbesartan) group (0.2% adenine diet + 20 mg/kg irbesartan by gavage). Each group was administered once daily for 4 consecutive weeks [16,23]. Twelve hours after the last administration, the animals were anesthetized with isoflurane, and their blood and kidneys were collected. A double-blinding strategy was applied in the present study to avoid bias. Independent researchers completed animal randomization and coding, and the operators for drug administration, sample collection, outcome assessment (pathological staining, ELISA, Western blot), and data analysis were all masked to group allocation until the final statistical analysis was finished.

#### 4.2.2. Sample Collection and Processing

Mice were anesthetized with 4% isoflurane in an induction chamber until loss of consciousness, and then maintained at a concentration of 1–3% via a mask. Once the toe pinch reflex disappeared for more than 60 s, breathing became deep and slow, and there was no limb contraction or respiratory change upon strong pinching, the head was fixed, and the jugular vein was gently compressed to engorge. A capillary tube was inserted into the inner canthus of the eye to collect blood, with the volume reaching 20–30% of the total blood volume (0.5–0.8 mL for 25 g mice). Immediately after blood collection, euthanasia was performed (under deep anesthesia, rapid cervical dislocation), and waiting for natural death due to blood loss was prohibited. Death was confirmed when breathing and heartbeat were observed to have stopped for at least 2 min and the pupils were dilated and fixed. The left kidney was collected and fixed in paraformaldehyde for histopathological examination, while the right kidney was rapidly frozen in liquid nitrogen and stored at −80 °C for molecular biological experiments.

#### 4.2.3. Renal Histopathological Examination

The renal tissues fixed with paraformaldehyde were dehydrated and embedded, and then 4 μm sections were prepared. After dewaxing and rehydration, the sections were stained with HE and Masson’s trichrome staining, respectively.

#### 4.2.4. Immunohistochemical (IHC) Staining

The renal tissues were sectioned, dewaxed, rehydrated, subjected to antigen retrieval, had endogenous peroxidase activity blocked, and were incubated with goat serum at room temperature for blocking. Then, the sections were incubated with primary antibodies against α-SMA and E-cadherin at 4 °C overnight, washed with PBS, incubated with the corresponding secondary antibodies at room temperature for 1 h, washed again with PBS, developed, counterstained with hematoxylin for the nuclei, dehydrated, and mounted for microscopic observation.

#### 4.2.5. Enzyme-Linked Immunosorbent Assay

The activities of SOD and the levels of MDA in renal tissues, the contents of IL-1β, IL-6, TNF-α, serum creatinine and urea nitrogen in serum, and the levels of IL-1β and IL-6 in cells were detected using enzyme-linked immunosorbent assay kits. Three technical replicates were set for each sample.

#### 4.2.6. Western Blot Analysis

For Western blot analysis, HK-2 cells and renal tissue specimens were processed in ice-cold lysis buffer supplemented with inhibitors, homogenized for 2 h, and cleared by centrifugation (12,000 rpm, 20 min, 4 °C). Total protein content was measured by the BCA method, and normalized samples were separated on 10% SDS-polyacrylamide gels before electrotransfer to PVDF membranes. The membranes were blocked with 5% skim milk for 2 h at room temperature, then probed overnight at 4 °C with primary antibodies against EGFR, p-EGFR, SRC, p-SRC, PI3K, p-PI3K, AKT, p-AKT, p65, and p-p65. After washing with TBST, blots were incubated with secondary antibodies for 2 h at room temperature, and protein bands were detected.

### 4.3. Network Pharmacology Analysis

The component targets were screened using the Swiss Target Prediction database (https://www.swissadme.ch/) with a score > 0.1 as the criterion. The CKD-related targets were retrieved from the Gene Cards database (https://www.disgenet.org/) by searching with the keyword “Chronic Kidney Disease”. A Venn diagram was drawn using the bioinformatics tool MicroBioInfo (https://www.bioinformatics.com.cn) to obtain the intersection targets of drugs and diseases. A protein–protein interaction (PPI) network was constructed using the STRING database (https://cn.string-db.org/) with a confidence score > 0.7 to screen the core targets. Gene ontology (GO) enrichment analysis and Kyoto Encyclopedia of Genes and Genomes (KEGG) pathway enrichment analysis were conducted using the Metascape database (https://www.metascape.org/).

### 4.4. Transcriptome Analysis

Total RNA was extracted from mice kidney tissues, and the extracted product was detected by ESilent 4150 TapeStation to confirm the size and integrity of the extracted product. mRNA was enriched using Oligo (dT) magnetic beads, and non-coding RNAs such as rRNA were removed. The mRNA was fragmented and reverse-transcribed into cDNA using reverse transcriptase. Sequencing adapters were ligated to both ends of the cDNA and amplified to form a library. The library was purified using magnetic beads to remove unnecessary impurities, and then quantified and normalized. The DNB was prepared by mixing. The DNB was loaded onto the high-throughput sequencing platform DNBSEQ-T7RS, and PE150 was selected for paired-end sequencing. Gene expression quantification was performed based on the alignment results, and differential gene analysis was conducted based on the gene expression quantification results.

### 4.5. Statistical Analysis

Data were expressed as the mean ± standard deviation and analyzed by GraphPad Prism 8.0.2 software (Dotmatics). A Shapiro–Wilk test was used to test for normal distribution, and Levene’s test was used to test for homogeneity of variance. The data that met the criteria were analyzed by one-way ANOVA and Tukey’s test, and the data that did not meet the criteria were analyzed by the Kruskal–Wallis H test with Dunn’s post hoc test for pairwise comparisons. *p* < 0.05 was considered to indicate a statistically significant difference.

## 5. Conclusions

In summary, this study not only experimentally confirmed the renal protective potential of ES in the adenine-induced AKI-to-CKD transition model through prophylactic co-administration, but also, through innovative multi-omics technology integration, discovered its molecular mechanism of action on the EGFR/SRC/PI3K/AKT/NF-κB signaling axis. This finding enriches the understanding of the mechanism by which ES intervenes in adenine-induced renal injury and prevents the AKI-to-CKD transition.

However, this study still has certain limitations. Although we verified the protein level changes at key nodes through Western blot and IHC, the direct physical binding mode of ES with EGFR or SRC molecules still needs to be further confirmed through techniques such as molecular docking simulation and surface plasmon resonance (SPR). Additionally, future research should further explore the selective regulatory effects of ES on other renal cell types (such as fibroblasts and macrophages) to comprehensively analyze its regulatory network in the complex renal microenvironment. This study provides strong scientific support for the development of ES as a new candidate drug for prevention of AKI-to-CKD transition and demonstrates the great potential of natural products in modern renal injury prevention and intervention.

## Data Availability

The data presented in this study are available on request from the corresponding author due to institutional data management policies and the need to protect ongoing research projects based on the present dataset.

## Abbreviations

The following abbreviations are used in this manuscript:

CKD	Chronic kidney disease
ES	Esculetin
HE	Hematoxylin–eosin staining
IHC	Immunohistochemical staining
SCr	Serum creatinine
BUN	Blood urea nitrogen
IL-1β	Interleukin-1 beta
IL-6	Interleukin-6
TNF-α	Tumor Necrosis Factor-α
MDA	Malondialdehyde
SOD	Superoxide dismutase
EMT	Epithelial–mesenchymal transition
α-SMA	Alpha-smooth muscle actin
EGFR	Epidermal Growth Factor Receptor
SRC	Src Proto-Oncogene
PI3K	Phosphatidylinositol 3-Kinase
AKT	Protein Kinase B
p65	Nuclear Factor κB p65 Subunit
p-	Phosphorylation
AKI	Acute kidney injury
AKI-to-CKD transition	Transition from acute kidney injury to chronic kidney disease
